# Heparan Sulfate, Chondroitin Sulfate, and Keratan Sulfate as Regulators of Tumor Malignancy

**DOI:** 10.3390/ijms27209229

**Published:** 2026-10-17

**Authors:** Yuki Ohkawa, Naoyuki Taniguchi

**Affiliations:** 1 Department of Glyco-Oncology and Medical Biochemistry, Next-Generation Precision Cancer Research Center, Frontier Exploratory Research Domain, Osaka International Cancer Institute, 3-1-69 Otemae, Chuo-ku 541-8567, Osaka, Japan; 2 Department of Molecular Oncology, Affiliated Graduate School of Medicine, The University of Osaka, 2-2 Yamadaoka, Suita 565-0871, Osaka, Japan

**Keywords:** tumor, glycocalyx, heparan sulfate, chondroitin sulfate, keratan sulfate

## Abstract

All mammalian cells, which include cancer cells, express sugar chains on their cell surfaces. These sugar chains bind with proteins, lipids, and RNAs. These chains consist of as few as two or three monosaccharides in the shortest chains, and as many as several hundred to a thousand monosaccharides in the longest chains. The chains appear to have a multilayered structure, which makes cells appear to be covered in a coating of sugar. The expression patterns and levels of these sugar chains, also known as the glycocalyx, vary greatly depending on the cell or tissue, and are therefore thought to perform unique localized biological functions. Furthermore, changes in the glycocalyx changes in conjunction with the onset of disease are believed to be closely related to the development of pathological conditions. In this study, we focus on glycosaminoglycans such as heparan sulfate, chondroitin sulfate, and keratan sulfate—relatively long chains of glycans that constitute the glycocalyx—and introduce their biological functions and potential applications in cancer treatment.

## 1. Introduction

A wide variety of glycans are expressed on the surfaces of mammalian cells, such as *N*-linked and *O*-linked glycans, glycolipids, proteoglycans, and GPI (glycosyl-phosphatidyl-inositol)-anchored proteins [1]. The glycans are predominantly bound to proteins, while RNAs are also known to be modified with *N*-linked glycans [2]. These glycans are biosynthesized by the sequential linking of monosaccharides such as glucose (Glc), mannose (Man), *N*-acetylglucosamine (GlcNAc), and sialic acid (Sia) through the action of glycosyltransferases. While the expression of sugar chains is physiologically controlled by the function of glycosyltransferases, it is also regulated by factors such as the amount of sugar nucleotides, organelle pH, and the activity of glycolytic enzymes; consequently, a research approach based on modern genomic analysis is difficult to apply, although such an approach currently plays a central role in current biology [3]. Furthermore, glycans are molecules of extremely high diversity with more than 10^9^ possible glycan structures based on combinations of monosaccharides [4]. This is why the identification and analysis of target glycans does not always proceed as expected.

As described above, the expression of glycans (glycocalyx) in cells and tissues is strictly regulated, but uncontrolled changes in their expression are closely related to the onset of disease and to the progression of pathological conditions [5]. Because glycans are expressed on the cell surface, they serve as the first point of entry for numerous forms of extracellular stimuli: ligand stimulation from growth factors, cytokines, and chemokines; stimulation from the extracellular matrix mediated by cell adhesion molecules involved in migration; and even mechanical stimulation associated with cell stretching and contraction. Abnormal glycan expression affects responsiveness to these stimuli, which alters intracellular signaling and modulates gene expression profiles, significantly contributing to the determination of cellular fate. Given these facts, elucidating the mechanisms of biological phenomena and disease onset involving glycans will be instrumental in developing glycan-targeted therapies that should no doubt become exponentially more important in the future.

Such a background explains why research into the involvement of glycans in cancer has been conducted for many years. It is noteworthy that many of the current and developing tumor markers are glycans and glycoproteins, which are referred to as cancer-associated glycans [6,7]. One of the most well-known tumor markers, CA19-9 (Carbohydrate Antigen 19-9), is a glycan known as sialyl Lewis a (sLe^a^). CA19-9 was first identified in the blood of patients with colorectal carcinoma [8,9]. In recent years, CA19-9 has been used for the diagnostic support of gastrointestinal cancers such as colorectal, gastric, and pancreatic cancers. It is important to note that CA19-9 not only functions as a marker but also abnormally activates EGFR (epidermal growth factor receptor) signaling, which contributes to tumorigenesis and an enhancement of malignant tumor characteristics [10]. Another cancer-associated glycan that has recently been under intense investigation is ganglioside GD2. GD2 is a glycosphingolipid consisting of a glucose (Glc), a galactose (Gal), an *N*-acetylgalactosamine (GalNAc), and two sialic acids bound to ceramide [11]. While GD2 is expressed at very low levels in normal skin and normal adult central nerve systems, an abundant and heterogenous expression of GD2 is often observed in melanomas, glioblastomas, and neuroblastomas. Therefore, GD2 is considered a favorable target for cancer immune system regimens such as chimeric antigen receptor (CAR) T-cell therapy [12,13]. As with CA19-9, the molecular functions of GD2 that enhance tumor malignancy have also been investigated by several research groups —including ours. For example, we know that GD2 strengthens cell signaling that is generated via growth factor receptors and integrins by modulating the lipid raft integrity of cancerous cells. When clustered in lipid rafts by GD2, cancerous cells transduce stronger downstream signaling via means such as the Src family kinase pathway, the phosphatidylinositol 3′-kinase (PI3K)/AKT pathway, and the focal adhesion kinase (FAK) pathway. Such signaling increases malignant properties such as rapid cell growth and enhanced cell invasiveness [14].

CA19-9 and GD2 are made up of short glycan chains formed with several monosaccharides. These complexes possess a significant level of biological importance, but to consider the function of the glycocalyx of cancerous cells, this review is focused on the long glycan chains expressed as glycosaminoglycans, which are identified by disaccharide repeating units and classified as heparan sulfate, chondroitin sulfate, keratan sulfate, dermatan sulfate, and hyaluronic acid. With the noted exception of hyaluronic acid, the disaccharides in these glycosaminoglycans are highly sulfated. It is common knowledge that the structural features and negative charges of sulfation groups characterize their biological functions. Therefore, their functions are deeply involved in the regulation of malignant tumor phenotypes. A proper review of the functions of the glycocalyx must focus on the biological functions of heparan sulfate, chondroitin sulfate, and keratan sulfate, all of which contribute to the malignancy properties of cancer. Because the glycan chains of glycosaminoglycans are much longer than those of *N*-glycans and *O*-glycans, their characteristic modulation exerts greater impact on the glycocalyx. There exist five types of glycosaminoglycans, but based on our research experience, we review only heparan sulfate, chondroitin sulfate, and keratan sulfate; the remaining two are dermatan sulfate and hyaluronic acid.

Glycosaminoglycans are very complex molecules. In addition to expression itself, glycan chain length, sulfation pattern, core protein behavior, and the shedding of glycan chains all tend to influence their biological functions and are involved in tumor malignancy. It is impractical to review and discuss all of these factors; therefore, for individuals who are not familiar with glycobiology, this study mainly focuses on the expression and regulation of cell signaling by glycosaminoglycans related to tumor malignancy.

## 2. Heparan Sulfate as a Cell-Signaling Modulator

**Figure 1:**
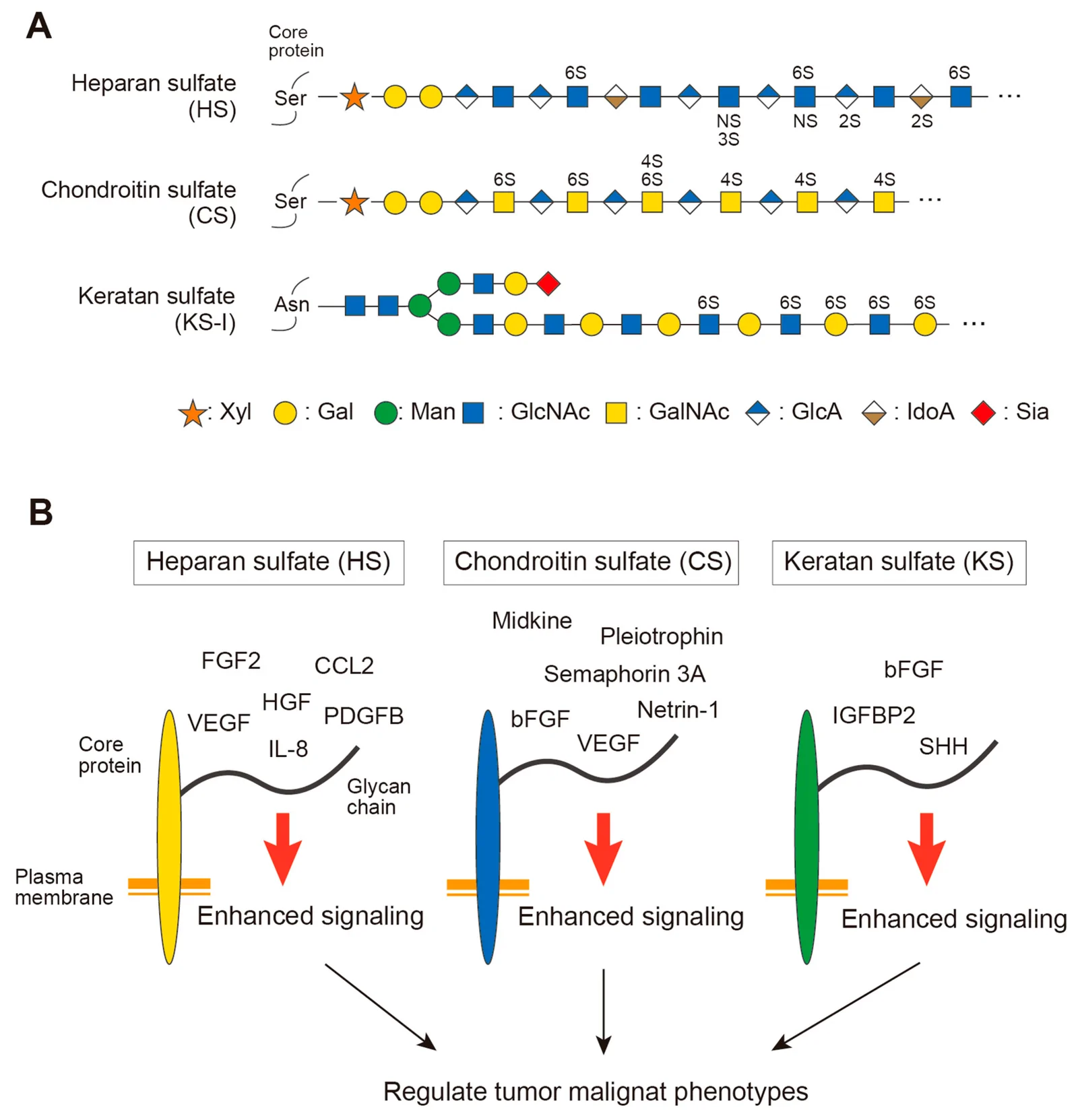
Glycosaminoglycans involved in tumor malignancy. (**A**) Representative glycan structures of heparan sulfate (HS), chondroitin sulfate (CS), and keratan sulfate (KS-I). Heparan sulfate and chondroitin sulfate are attached via a serine residue (Ser) in core proteins, while keratan sulfate (KS-I) is attached to an asparagine residue (Asn) in core proteins. The tetrasaccharide structure consisting of xylose–galactose–galactose–glucuronic acid (Xyl-Gal-Gal-GlcA) is shared by HS and CS. The disaccharide repeats Gal-GlcNAc of KS-I are generated at the *N*-glycan backbone. The designations 2S, 3S, 4S, and 6S indicate sulfation groups on carbon positions 2, 3, 4, and 6, respectively. NS indicates a sulfation group on the N-acetyl group. (**B**) Representative small molecules associated with heparan sulfate (HS), chondroitin sulfate (CS), and keratan sulfate (KS). Co-receptors HS, CS, and KS regulate cell signaling via their specific receptors, which results in the modulation of oncogenic phenotypes in cancer.

Heparan sulfate is a type of glycosaminoglycan (mucopolysaccharide), and the compound formed when heparan sulfate is attached to a protein (core protein) is referred to as a heparan sulfate proteoglycan. Heparan sulfate proteoglycans are formed when a tetrasaccharide structure consisting of xylose–galactose–galactose–glucuronic acid (Xyl-Gal-Gal-GlcA) is attached to the serine (Ser) residue of the core protein, which is followed by an extension made up of a disaccharide repeating unit consisting of uronic acid (glucuronic acid or its epimer, iduronic acid) (GlcA or IdoA) and *N*-acetylglucosamine (GlcNAc) (Figure 1A) [15,16]. This disaccharide repeating unit can exceed approximately 500 units in the longest chains. The physical size per molecule contributes to its status as one of the major components of the glycocalyx. Furthermore, as the name “sulfate” suggests, the sugar chains within the repeating structure of this disaccharide are frequently sulfated, which contributes to the generation of a strong negative charge. This negative electric field is thought to be one of the mechanisms underlying the ability to capture the growth factors described later. Furthermore, the core proteins of heparan sulfate proteoglycans have been identified as Syndecan and Glypican (membrane-bound types), Serglycin (secreted type), Perlcan, Agrin, and collagen type XVIII (extracellular matrix types) [17]. The reason why only these proteins are targeted for modification with heparan sulfate remains unknown. Further studies are needed to answer this question.

In 1991, groups led by Rapraeger and Yayon published the first report describing the binding of heparan sulfate to FGF2 (fibroblast growth factor 2) [18,19]. The binding of heparan sulfate to the heparin-like binding site of FGF2 is known to stabilize the structure of the FGF2-FGFR (fibroblast growth factor receptor) complex and to generate a stronger FGFR signal. In particular, it has been reported that sulfation of the carbon at position 6 of *N*-acetylglucosamine (GlcNAc) within the heparan sulfate disaccharide repeats is known to be a necessary factor in the binding of heparan sulfate to FGF2 and FGFR [20]. Following this discovery, it became clear that several secreted proteins bind to heparan sulfate: HGF (hepatocyte growth factor), VEGF (vascular endothelial growth factor), IL-8 (interleukin-8), CCL2 (C-C motif chemokine ligand 2), and Wnt (Figure 1B). Based on these findings, heparan sulfate was found to function as a co-receptor for the receptor signaling of growth factors, cytokines, and chemokines [21].

FGFR amplifications and mutations are often observed in many cancers, and the FGFR signal is considered to be deeply related to malignant tumor phenotypes [22]. As described above, heparan sulfate enhances FGFR signal transduction by working as a co-receptor, which is why heparan sulfate is considered a possible target for suppressing the oncogenic signals that arise from FGFR. On the other hand, VEGF is also a possible therapeutic target due to its relationship to heparan sulfate. Angiogenesis is essentially for growing cancer tissues, and VEGF is one of the factors that generate nascent blood vessels and enhance vascular permeability [23]. Cancerous cells express VEGF and bind with the VEGFRs (vascular endothelial growth factor receptors) expressed on endothelial cells, which enhances the cell division of endothelial cells for the generation of blood vessels [24]. The binding affinity of VEGF to heparan sulfate has been clearly demonstrated [25], and evidence shows that heparan sulfate potentiates the angiogenesis mediated by VEGFR signaling [26]. Therefore, inhibiting heparan sulfate is a possible strategy to weaken VEGFR signaling and block angiogenesis in cancers. Notably, heparan sulfate also induces vascular hyperpermeability by regulating VEGFR signaling [27], which contributes to the improvement of oxygen deficiency in cancer tissues and supports the movement of cancerous cells into vessels in the early stages of metastasis. An interesting report published by Lima and colleagues described how sulfated glycans such as heparan sulfate are expressed on exosomes and tend to gather CCL2 in the tumor microenvironment [28]. These exosomes are derived from cancerous cells and are transferred to the other organs where they are recognized by the receptor CCR2 (C-C motif chemokine receptor 2) that is expressed on NK cells and MDSCs (myeloid-derived suppressor cells), which are involved in the establishment of a pre-metastatic niche.

In recent years, we have also reported on the function of heparan sulfate in brain tumors (gliomas). Knocking out the *Ext-1* (*Exostosin-1*) gene, which is responsible for the biosynthesis of the disaccharide repeat structure of heparan sulfate, results in a loss of heparan sulfate expression. By comparing these cells with control cells (*Ext-1* wild-type), we demonstrated that PDGFB (platelet-derived growth factor B) is captured on the cell surface in a manner dependent on heparan sulfate expression [29]. We described how the capture of PDGFB leads to the acquisition of malignant phenotypes by enhancing the PI3K (phosphatidylinositol 3-kinase)/AKT and MAPK (mitogen-activated protein kinase) signaling pathways, which are transmitted via its receptor, PDGFRα (platelet-derived growth factor receptor α). This co-receptor function of heparan sulfate for PDGFRα signaling confers glioma cells with resistance to erlotinib, which is an EGFR (epidermal growth factor receptor) inhibitor.

As this evidence suggests, heparan sulfate is a possible target for cancer therapy. The silencing of biosynthesis-responsible genes such as *EXT-1* and *EXT-2* would be a way to accomplish inhibition, and a compound that is currently under development, referred to as Surfen (*bis*-2-methyl-4-amino-quinolyl-6-carbamide), is a unique antagonist of heparan sulfate. In 2008, Schuksz and colleagues were the first to identify Surfen and additionally demonstrated that it specifically inhibits FGF2 and VEGF binding to heparan sulfate [30]. In another study, the molecular domain of Surfen that is responsible for its binding inhibition was defined, and the derivatives were also developed to enhance its inhibitory ability [31]. Additionally, the function of heparan sulfate as a signaling cofactor is known to be regulated by its sulfate groups, and in particular, the sulfation of the carbon position 6 of *N*-acetylglucosamine (GlcNAc) is necessary for the binding of heparan sulfate to FGF2 and FGFR, as described above [20]. This sulfation is carried out by heparan sulfate 6-*O*-sulfotransferase (HS6ST) in the Golgi apparatus within cells, while its frequency is regulated by sulfatases, which are desulfation enzymes. While most sulfatases are localized to intracellular lysosomes, SULF-1 and SULF-2 have been identified as sulfatases that function in extracellular regions [32]. The involvement of SULF-2 in glioma malignancy has been demonstrated, and the regulation of heparan sulfate function via the desulfation of enzymes could be another method of cancer treatment. In addition, recent reports have shown that the cell surface conjugates of glycoRNA and RNA-binding protein inhibits heparan sulfate-mediated VEGF-A signaling [33]. Given these facts, it should be apparent that glycoRNA could also be a target for regulating the function of heparan sulfate as a signaling modulator.

## 3. Chondroitin Sulfate as a Cell-Signaling Modulator

Chondroitin sulfate is another type of glycosaminoglycan, which is characterized by a disaccharide repeat of glucuronic acid (GlcA) and *N*-acetylgalactosamine (GalNAc) [34]. It is noteworthy that chondroitin sulfate and heparan sulfate both have a tetrasaccharide structure (Xyl-Gal-Gal-GlcA) that links them to core proteins, but of these two glycosaminoglycans, only the biosynthesis of chondroitin sulfate can be critically regulated by targeting the tetrasaccharide structure with GalNAc transferases [chondroitin sulfate *N*-acetylgalactosaminyltransferases 1 and 2 (CSGALNACT1/2)] [35]. Additionally, the functional meanings of the sulfation patterns of chondroitin sulfate disaccharide have been studied more than those of heparan sulfate. Many reports have documented the specific biological functions in these sulfation patterns that are classified into four units: CS-A (carbon position 4 of GalNAc with a sulfation group), CS-C (carbon position 6 of GalNAc with a sulfation group), CS-D (carbon position 2 of GlcA and carbon position 6 of GalNAc with sulfation groups), and CS-E (carbon positions 4 and 6 of GalNAc with sulfation groups) [36]. Of these patterns, CS-E from bovine trachea shows a strong binding affinity to FGF2, BDNF (brain-derived neurotrophic factor), and IL-10 (interleukin 10) [37,38,39]. Similarly to the results cited in this review, Ueoka and colleagues found that CS-E strongly binds to Midkine, which is a small multi-functional secreted protein [40]. Additionally, the sulfation pattern of chondroitin sulfate is involved in the disease state and in its development. For instance, CS-C is known to exert protective functions against skin inflammation via the suppression of EGFR signaling in keratinocytes, which is associated with psoriasis [41]. In neuronal cells, chondroitin sulfate has been identified as a novel substrate for the receptor-type protein tyrosine phosphatase PTPRσ [42]. Following stimulation, particularly that from CS-E, PTPRσ dephosphorylates cortactin and forms a dystrophic endball, and the result is strong inhibition of neuronal regeneration [43]. Additionally, Miyata and colleagues focused on the ratio of the sulfation at carbon position 4 to sulfation at carbon position 6 of chondroitin sulfate in neuroplasticity. They clearly demonstrated that an increase in the 4-sulfation/6-sulfation ratio changes the properties of the perineuronal net and induces termination of the critical period for ocular dominance plasticity in the visual cortex of mice [44]. These data strongly suggest that the sulfation group in each of the carbon positions of chondroitin sulfate codes individual biological functions.

The core proteins that carry chondroitin sulfate have been identified. Aggrecan, which is also known as chondroitin sulfate proteoglycan 1 (CSPG1), is one of the most expressive proteoglycans in cartilage. According to its strong water-holding capacity, aggrecan is thought to be a molecule that allows for shock absorption in joints. Versican and neurocan are other types of core proteins, and are referred to as chondroitin sulfate proteoglycan 2 (CSPG2) and chondroitin sulfate proteoglycan 3 (CSPG3), respectively. They are known for their specific expression in central nerve tissues under development and for their involvement in the developing brain [45]—particularly in reports of the maturation of presynaptic neurons [46]. Other core proteins include brevican, phosphacan, and CD44. These also are expressed in central nerve tissues, which is the reason their neurological functions are popular topics of investigation [47]. On the other hand, as briefly described above, chondroitin sulfate captures several small molecules such as growth factors and works as a co-receptor for receptor signaling, which is similar to the function of heparan sulfate. The specific binding of chondroitin sulfate to midkine, pleiotrophin, semaphorin 3A, and netrin-1 has been identified, and this strongly suggests their involvement in neurological functions [48]. It is noteworthy that FGF2 and VEGF are also predicted to be chondroitin sulfate-binding proteins, and the regulation of FGFR and VEGFR signaling by chondroitin sulfate could be a target for cancer therapies (Figure 1B) [49,50]. A small compound that inhibits chondroitin sulfate proteoglycan by enhancing the biosynthesis of free chondroitin sulfate chains has been identified [51,52]. This is a compound that could potentially target cancerous cells by inhibiting the function of chondroitin sulfate as a co-receptor. Since this compound is not as specific to cancer cells as Surfen, an antagonistic inhibitor against heparan sulfate described above, it would induce dysregulation of glycosaminoglycans by inhibiting their physiological roles as side effects in normal cells and tissues.

## 4. Oncofetal Chondroitin Sulfate: A Unique Target for Cancer Therapies

The term “oncofetal” refers to the cellular characteristics of the proteins and antigens that are typically present under embryonic and fetal development, but which could become abnormally re-expressed in adult tissues during oncogenesis [53]. Oncofetal proteins/antigens are considered ideal targets for cancer therapy due to the specificity of their expressions in cancers. For example, the oncofetal α-fetoprotein is normally produced only in fetal liver and umbilical vesicles, but not in healthy adults [54]. Therefore, it is interesting that the re-expression of α-fetoprotein is observed in many hepatocellular carcinoma patients. This protein is detected in the peripheral blood, which is why α-fetoprotein has been developed as a cancer biomarker that is available for clinical use [55]. Carcinoembryonic antigen (CEA) is another example of an oncofetal antigen [56] that is broadly used as a tumor marker in clinical settings [57]. In a healthy body, the concentration of CEA in the blood does not exceed 5 ng/mL [58], but its levels are mainly elevated in patients with colorectal, gastric, lung, and breast cancers. CEA is also known to function as an intercellular adhesion molecule [59], but its function as a tumor marker has increased in importance.

A concept of the characteristics of oncofetal chondroitin sulfate was first proposed by Salanti and colleagues in 2015 [60]. They found that malarial protein VAR2CSA specifically binds to chondroitin sulfate in a distinct sulfation pattern that is expressed in the placenta. In further studies, a high sulfation ratio, approximately 85%, at carbon position 4 of chondroitin sulfate indicates a CS-A unit and defines a distinct sulfation pattern that contributes to determination of their unique expression in colorectal carcinomas [61]. This high sulfation ratio at carbon position 4 of chondroitin sulfate is not observed in normal human adult tissues, with the noted exception of the placenta, which strongly indicates its oncofetal character. In addition to colorectal carcinomas, the expression of oncofetal chondroitin sulfate is available for the detection of circulating cancerous cells in the blood, and it is found in cancers such as lung, pancreatic, hepatic, and prostate [62]. Trials are underway to develop a diagnostic method that will target oncofetal chondroitin sulfate [63]. Additionally, several functional aspects of oncofetal chondroitin sulfate have been revealed. For example, oncofetal chondroitin sulfate is known to be required for sufficient integrin signaling. This was discovered via analysis with a recombinant VAR2CSA protein, which potentially indicates that oncofetal chondroitin sulfate potentially enhances the cell migration and invasion activities that are associated with metastasis [64]. The use of oncofetal chondroitin sulfate to indicate CS-A in immune systems has also been suggested. CS-A enhances T cell differentiation into CD4-positive T cells through the signal transduction and activation of transcription 3 and 4 (STAT3 and 4), which results in the upregulation of interleukin-17 (IL-17) and interleukin-23 (IL-23) [65]. In addition, another group reported that CS-A blocks C-C motif chemokine ligand 5 (CCL5) responses in monocytes by binding with it, reducing the adhesion of monocytes to endothelial cells along with their migration [66]. These data also indicate the involvement of oncofetal chondroitin sulfate in tumor immunity. Taken together, these established facts suggest that oncofetal chondroitin sulfate would be a beneficial target for cancer diagnosis and treatment. However, further determination of the structural and functional mechanisms is needed.

## 5. Keratan Sulfate in Tumor Malignancy

As with other glycosaminoglycans, keratan sulfate is also a type of glycosaminoglycan. Keratan sulfate proteoglycans are broadly classified into three types: KS-I, KS-II, and KS-III [67]. In KS-I, an *N*-glycan backbone is formed on the asparagine (Asn) residues of the core protein, which is followed by the extension of a disaccharide repeating unit consisting of galactose (Gal) and *N*-acetylglucosamine (GlcNAc) (Figure 1A). In KS-II, an *O*-glycan backbone of the type 2 core is formed on either the serine or threonine residue (S/T) of the core protein, as well as by the extension of a Gal-GlcNAc disaccharide repeating structure. KS-III has a structure in which Gal-GlcNAc disaccharide repeating units are extended via α-mannosidic linkages to either the serine or threonine residue (S/T) of the core protein [68]. Although the binding patterns of core proteins for keratan sulfate are diverse, it is important to note that all of them possess a Gal-GlcNAc disaccharide repeating unit. As for the core proteins of keratan sulfate, podocalyxin has been identified as a transmembrane protein [69], lumican as a secreted and extracellular matrix-associated protein [70], and aggrecan as a secreted protein [71,72]. Keratan sulfate is thought to have the ability to bind to secreted proteins such as FGF2, IGFBP2 (insulin-like growth factor binding protein 2), and SHH (sonic hedgehog), which suggests that it functions as a co-receptor in the receptor signaling pathways of these ligands (Figure 1B) [73,74].

To date, the facts connecting keratan sulfate to cancer remain limited. A few types of cancers such as pancreatic cancer, melanoma, and glioma derived from astrocytic lineage cells have been reported as keratan sulfate high-expression phenotypes [75,76,77]. Intracytoplasmic accumulation of keratan sulfate was also reported as a feature of granular cell tumors [78]. Similar with other glycosaminoglycans, sulfation levels and sulfation patterns of keratan sulfate glycan chains would be related to cancer development due to the fact that low-sulfated keratan sulfate is expressed in non-mucinous ovarian cancer [79]. Since these studies mainly analyzed the expression of keratan sulfate, further studies are needed to understand the molecular functions of keratan sulfate with respect to tumor malignancy. Additionally, there are several reports describing the connections between keratan sulfate’s core proteins in cancers. For example, lumican is expressed and secreted from human melanoma cells [80]. Additionally, lumican is known to suppress migration activity via blocking MMP-14 (matrix metalloproteinase-14) and to inhibit tumors grown in mice [81,82]. Another group of researchers reported that lumican regulates cell invasion activity through p120 catenin [83]. On the other hand, another keratan sulfate core protein, podocalyxin, was reported as a key enhancer of the growth and metastatic ability of breast cancer cells [84]. Under the progression of epithelial–mesenchymal transition (EMT), podocalyxin is upregulated and enhances the podocalyxin–ezrin signaling axis in breast and pancreatic cancer cells [85]. Podocalyxin also is reported to increase the invasiveness of cells by binding with galectin-3 on the cell surface [86]. Although CAR-T cell therapy targeting podocalyxin is currently under development [87], unfortunately, these studies did not analyze the functions of the keratan sulfate glycan chain, which is likely involved in the function of lumican in those cancers.

In recent years, our group has demonstrated the ectopic expression of keratan sulfate in melanoma. Although no correlation has been observed between keratan sulfate expression and skin cancer subtypes, keratan sulfate expression was observed in more than half of the analyzed specimens [76]. Furthermore, analysis of keratan sulfate function using human melanoma cell lines revealed that keratan sulfate significantly enhances cell invasion activity by potentiating the function of the cell adhesion molecule integrin. Keratan sulfate is thought to promote integrin clustering by interacting with integrins either directly or indirectly. Another research group also reported the involvement of keratan sulfate in cell adhesion [88]. Although elucidating the detailed mechanism remains a future challenge, it is highly likely that keratan sulfate expression could be utilized to assess the invasiveness of skin cancer. These results are of interest as a unique function of keratan sulfate. Thus, changes in the glycocalyx resulting from keratan sulfate expression are thought to act on the regulation of cell motility and cell invasion through the control of integrin function.

## 6. Keratan Sulfate Disaccharide as Anti-Inflammatory Glycomimetics: An Example of Immune Regulation

**Figure 2:**
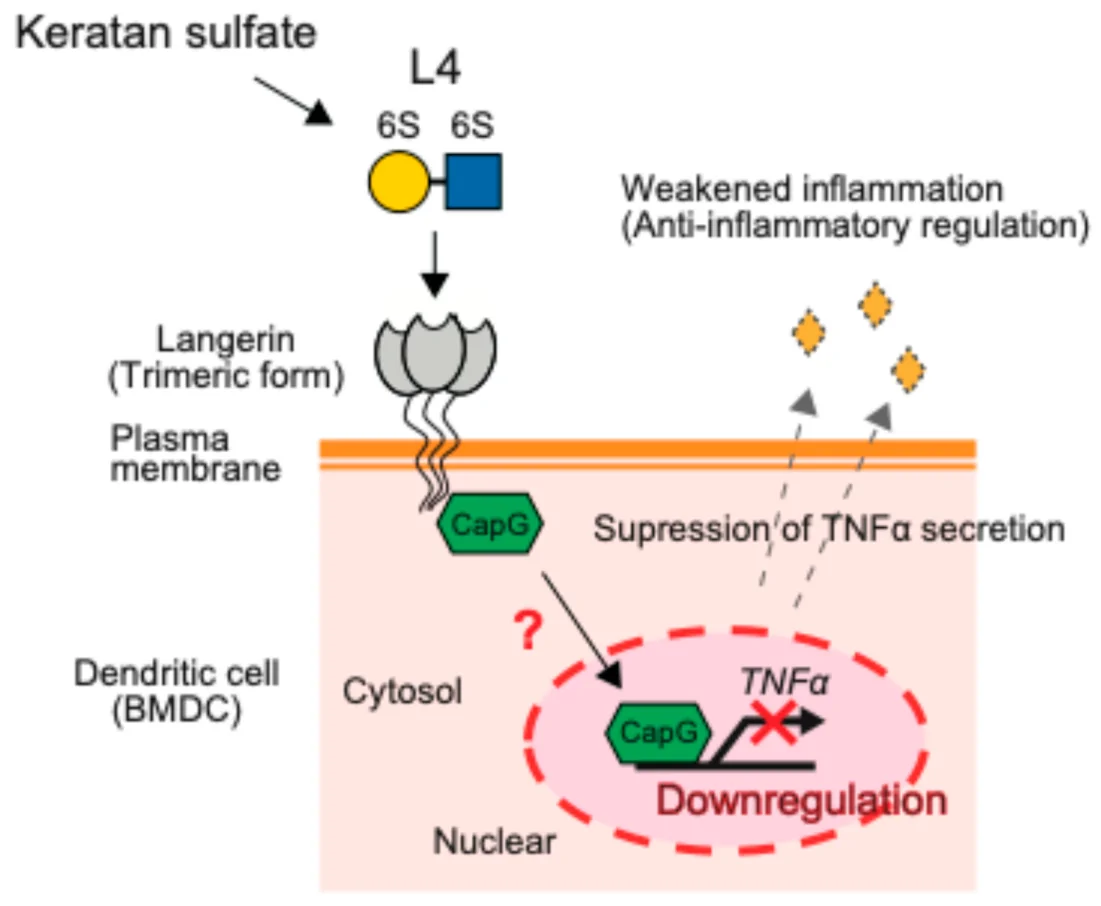
Keratan sulfate-based disaccharide L4 as an anti-inflammatory glycomimetic. L4 [a disaccharide consisting of galactose and *N*-acetylglucosamine (GlcNAc) with sulfation groups on carbon position 6] is identified as a glycan ligand for the C-type lectin receptor Langerin. After binding L4, a macrophage-capping protein (CapG) associated with the cytosolic region of Langerin translocates into the nucleus. CapG binds to the promoter region of the *tumor necrosis factor-α* (*TNFα*) gene and downregulates it, which results in the suppression of TNFα secretion. Such regulation of immunity by L4 could potentially be applied to develop a novel technology in cancer immunotherapies.

In addition to the regulation of integrin function, we recently demonstrated how the administration of keratan sulfate—specifically the disaccharide (Gal-GlcNAc, referred to as L4; both monosaccharides have a sulfation group at carbon number 6) that constitutes the repeating sugar chain structure of keratan sulfate—significantly improves inflammation in mouse models of emphysema [89]. L4 acts as a ligand for Langerin, one of the C-type lectin receptors expressed on dendritic cells [90,91]; in addition, we recently identified macrophage-capping protein (CapG), which is an intracellular protein that interacts with Langerin. Analysis using bone marrow-derived dendritic cells (BMDCs) derived from mouse bone marrow has revealed how the binding of L4 to Langerin induces the nuclear translocation of CapG, and that CapG suppresses the gene expression of the inflammatory cytokine TNF-α (tumor necrosis factor α) [92]. The alleviation of inflammatory pathology observed in a mouse model of emphysema is known to be due to the anti-inflammatory action of L4 via the molecular mechanism Langerin → CapG → suppression of TNF-α expression (Figure 2). These results were obtained by exogenously administering keratan sulfate disaccharide L4, and it remains unclear whether endogenous keratan sulfate exerts a similar effect. Verifying whether changes in the glycocalyx caused by keratan sulfate expression contribute to the regulation of inflammation remains a topic for future research. Furthermore, researchers have suggested that keratan sulfate could regulate the functions of tumor-associated macrophages and other cells, and we look forward to new insights in the field of cancer research. Since these data are deemed to be somewhat speculative, steady and constructive studies are required in the future.

Biomimetics is a field of science and technology that leverages the structures, functions, and production processes found in living organisms and in nature. Practitioners of this discipline then apply this knowledge to the development of new technologies and products [93]. Well-known examples include fans modeling the shape of butterfly wings, adhesive tape inspired by the adhesive properties of gecko feet, and competitive swimsuits designed to mimic the microstructure of shark skin. Within the field of biomimetics, the branch specifically focused on sugar chains is referred to as glycomimetics [94,95], and our research group is currently striving to develop an L4-based branch of glycomimetics. The multivalent effects of L4 inspired us to employ chemical synthesis techniques to prepare Triangle L4 (a trimer of L4) and Pendant L4 (a polymer of L4) and to analyze their binding affinity for Langerin. We have demonstrated how these derivatives bind more strongly than the L4 monomer [91]. Additionally, we have also identified the manner in which a sulfation group on the *N*-acetylglucosamine (GlcNAc) residue of L4 is required for its binding to Langerin [91]. Based on this background, we will continue to devote efforts to the development of unique aspects of glycomimetics by analyzing the anti-inflammatory effects of these derivatives. It is noteworthy that inflammatory status is involved in tumor progression [96]; therefore, we believe that L4 and its derivatives could be used to modulate tumor status by studying and utilizing their immune regulation functions.

## 7. Putative Functions of Glycosaminoglycans in Non-Cancerous Cells in Tumor Microenvironment

Tumor tissues are generated not only with cancer cells but also with non-cancerous cells such as fibroblasts, endothelial cells, and several types of immune cells: macrophages, natural killer (NK) cells, and lymphocytes [97]. The above sections review the function of glycosaminoglycans mainly in cancer cells; however, glycosaminoglycans also play important roles in the non-cancerous cells of the tumor microenvironment.

Non-cancerous fibroblasts exist in tumor tissues and are defined as cancer-associated fibroblasts (CAFs). Interestingly, the specific ablation of heparan sulfate in CAFs has promoted tumor growth in a study using a mouse xenograft model [98]. In this study, however, the molecular functions of heparan sulfate in CAFs were not investigated well, although the ablation of heparan sulfate in CAFs were found to upregulate matrix metalloproteinase-7 (MMP-7), which clearly contributed to rapid tumor growth. On the other hand, the study showed how the expressions of heparan-6-*O*-endosulfatase 1 and 2 (SULF1 and SULF2) in CAFs are involved in malignant tumor phenotypes. SULF-1 promotes gastric cancer metastasis and anti-cancer drug resistance against cisplatin via the regulation of transforming growth factor-β receptor III (TGFBR3)-mediated transforming growth factor-β (TGF-β) receptor signaling [99], and SULF-2 enhances the invasiveness of head and neck squamous carcinoma cell lines in co-culture analyses [100].

Heparan sulfate and chondroitin sulfate works on the endothelial cells that potentially regulate tumor malignancy. For example, endothelial cell-derived heparan sulfate binds FGF2 and avoids its degradation by extracellular proteinases, which would enhance FGFR signaling in cancer cells [101]. Nagano and colleagues reported that chondroitin sulfate directly interacts with extracellular histones to prevent its toxicity in vascular endothelial cells [102]. Although further experiments demonstrating the relationships with tumor malignancy are needed, this mechanism would contribute to angiogenesis. Interestingly, heparan sulfate is also considered a mechanosensory element in endothelial cells [103]. Another study found that by impairing shear stress-related AMPK/FoxO1 signaling, the resultant ablation of heparan sulfate upregulates angiopoietin-2 in endothelial cells [104]. While that study was not focused on tumor behavior, the upregulation of angiopoietin-2 was found to potentially enhance angiogenesis in tumors.

On the other hand, many studies have described macrophage regulation by glycosaminoglycans. Heparan sulfate helps the binding and uptake of low-density lipoproteins (LDLs) in macrophages [105]. Additionally, heparan sulfate in macrophages is required to control their basal activation by maintaining type I interferon signaling, which is strongly related to sulfation levels of heparan sulfate [106]. The inhibitory functions of chondroitin sulfate are also reported. For example, addition of chondroitin sulfate inhibits NF-κB responses induced by lipopolysaccharide (LPS) or hyaluronan fragments in macrophages [107]. In a mouse model of Duchenne muscular dystrophy, chondroitin sulfate produced as chondroitin sulfate proteoglycan-4 (CSPG4) from macrophages inhibits nerve sprouting and modulates neural functions [108]. Keratan sulfate is also found in microglia/macrophages in nerve tissues, which regulates microglia/macrophage activation during spinal cord injury, autoimmune neuritis, and Wallerian degeneration [109,110,111].

Taken together, these findings suggest that glycosaminoglycans in non-cancerous cells are important regulators of the tumor microenvironment and could indirectly influence tumor progression by modulating intercellular communication, extracellular matrix organization, angiogenesis, and immune responses. However, compared with the extensive evidence accumulated for glycosaminoglycans in cancer cells, their cell type-specific functions in stromal and immune cells remain relatively poorly understood. In particular, the precise molecular mechanisms by which distinct glycosaminoglycan structures, sulfation patterns, and their associated proteoglycans regulate the phenotypes and interactions of non-cancerous cells in tumors will require further elucidation. Future studies focusing on the spatial and cell type-specific regulation of glycosaminoglycans will therefore be important to promote understanding of the complexity of the tumor microenvironment and could provide new opportunities for therapeutic intervention.

## 8. Other Molecular Aspects of Glycosaminoglycans Related to Tumor Malignancy

The co-receptor functions of glycosaminoglycans have been well studied, as described above. Other molecular aspects are emphasizes in this review in order to promote the recent advances in the understanding of the regulation of tumor malignancy. One of these aspects involves physical properties. Barnes and colleagues used alcian bule staining to reveal that mesenchymal-like glioblastoma is rich in glycosaminoglycans [112]. They also demonstrated that glycosaminoglycan-rich glioblastoma tissue is harder than glycosaminoglycan-plain glioblastoma. This hardness property regulates the integrin-focal adhesion kinase (FAK) signal, which enhances malignant properties such as strong cell migration and invasion activity and has promoted faster tumor growth in mouse xenograft models. Additionally, these researchers reported that physical properties often function as a physical shield against chimeric antigen receptor T-cells (CAR-T). Tumors such as pancreatic cancer possess abundant stroma that make them resistant to CAR-T therapy [113]. Since the stroma region is rich in glycosaminoglycans, the elimination of these glycosaminoglycans is considered a possible strategy to enhance the killing of cancerous cells by CAR-T. Notably, Caruana and colleagues clearly demonstrated how the removal of heparan sulfate in the stroma via the expression of heparanase, which is an enzyme that digests heparan sulfate, on CAR-T promotes the infiltration of tumors and anti-tumor activity [114]. There have been no critical studies on the targeting of chondroitin sulfate for CAR-T therapy, but it should be regarded as an ideal molecule, which is why fundamental studies are likely to already be in the start-up phase. Along with glycosaminoglycans, other types of glycans, such as the hyaluronic acids and high-branched *N*-glycans that are expressed on cell surfaces and in the stroma, are additionally reported as targets to enhance the effects of CAR-T therapy [115,116]. The combination of these data introduces other interesting molecular aspects of glycosaminoglycans with respect to the regulation of tumor malignancy.

**Figure 3:**
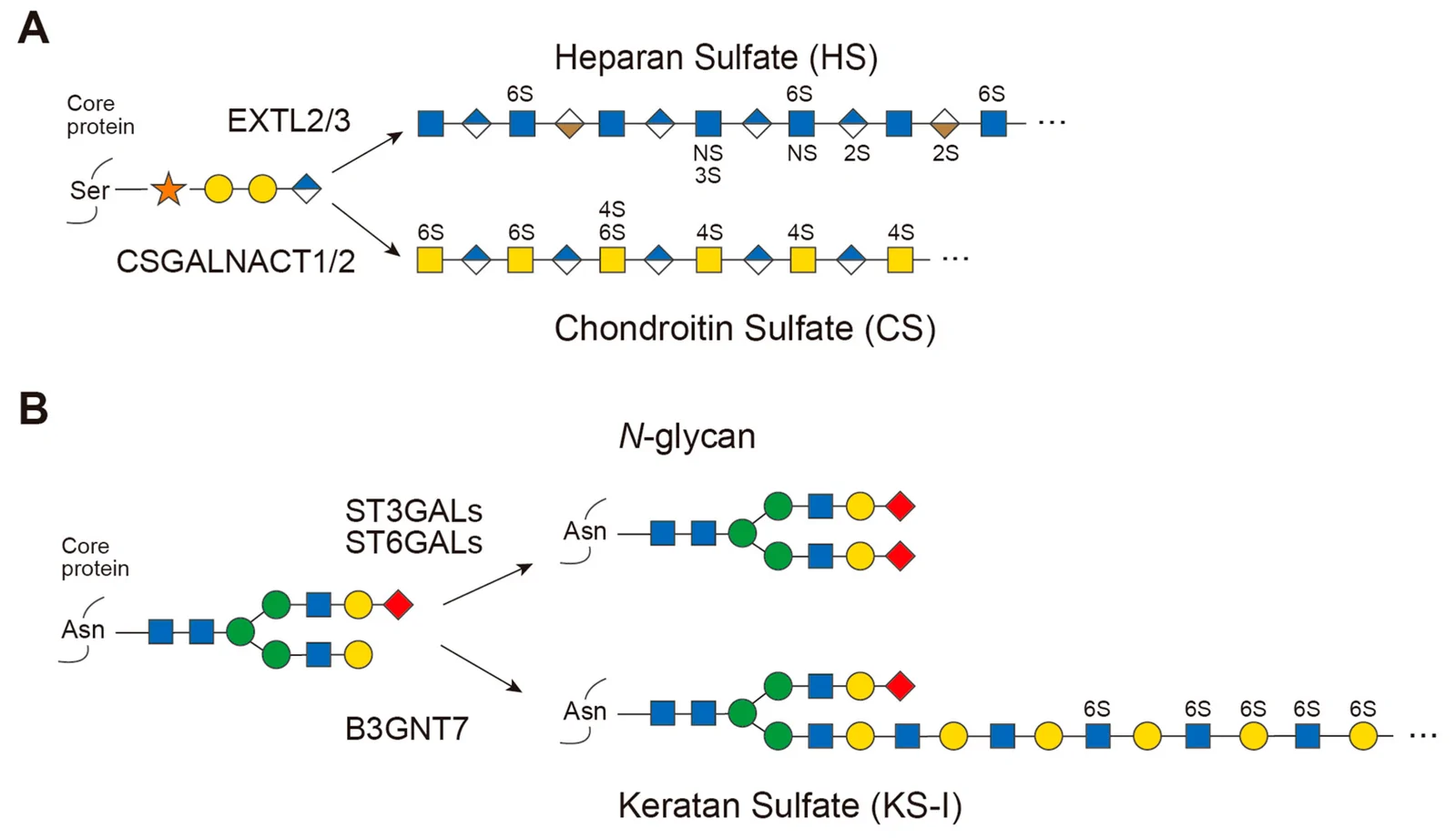
Competitive mechanisms for biosynthesis of glycosaminoglycans. (**A**) The tetrasaccharide structure consisting of xylose–galactose–galactose–glucuronic acid (Xyl-Gal-Gal-GlcA) is shared by HS and CS. When Exostosis-like 2 and 3 (EXTL2/3) predominantly work on the tetrasaccharide, heparan sulfate (HS) is generated. On the other hand, if chondroitin sulfate *N*-acetylgalactosaminyltransferase 1 and 2 (CSGALNACT1/2) primarily work on tetrasacharide, chondroitin sulfate (CS) is generated instead of HS. (**B**) A disaccharide repeat consisting of galactose (Gal) and *N*-acetylglucosamine (GlcNAc) of KS-I is generated on the *N*-glycan backbone. When β1,3-*N*-acetylglucosaminyltransferase 7 (B3GNT7) predominantly works on the backbone, keratan sulfate (KS-I) is generated. If either β-galactoside α-2,3-sialyltransferases (ST3GALs) or β-galactoside α-2,6-sialyltransferases (ST6GALs) function well, modification with terminal sialic acid predominantly occurs, and elongation of the disaccharide repeat of KS-I is not conducted.

Another notable machinery of glycosaminoglycan is the selective biosynthetic regulation between heparan sulfate and chondroitin sulfate. As described above, both glycosaminoglycans share an innermost tetrasaccharide structure. Exostosis-like 2 (EXTL2) and 3 (EXTL3) are enzymes involved in the production of heparan sulfate [117,118], and chondroitin sulfate *N*-acetylgalactosaminyltransferase 1 (CSGALNACT1) and 2 (CSGALNACT2) are enzymes involved in the production of chondroitin sulfate [119,120]. These enzymes compete for the tetrasaccharide as a substrate, and the type of glycosaminoglycan that is generated depends on the expression levels of these enzymes in the cells. Higher expression levels of either EXTL2/3 or CSGALNACT1/2 define the preferential expression of either heparan sulfate or chondroitin sulfate, respectively (Figure 3A). Cell signaling regulation is different between heparan sulfate-expressing cells and chondroitin sulfate-expressing cells, which is a factor of the differences in the binding affinities of growth factors and results in the determination of different cell fates. This would be an important point on which to focus when considering the regulation of tumor malignancy. Notably, Bång-Rudenstam and colleagues clearly demonstrated that biosynthetic switching from heparan sulfate to chondroitin sulfate confers resistance to ferroptosis in glioma cells [121]. On the other hand, selective biosynthetic regulation between keratan sulfate and *N*-glycan is another expected machinery of glycosaminoglycans. The structural backbone of KS-I is the same as that of *N*-glycans, as described above, and therefore, the predominant production of keratan sulfate is relayed to the expression of β1,3-*N*-acetylglucosaminyltransferase 7 (B3GNT7), which is an enzyme that transfers *N*-acetylglucosamine (GlcNAc) via a β1,3 linkage [122,123]. This enzymatic reaction is required for the first-step addition of GlcNAc residue and for the sequential elongation steps involving disaccharide repeats of keratan sulfate. Similar to the cases of heparan sulfate and chondroitin sulfate described above, predominancy for the generation of keratan sulfate is critical for the determination of cell fate (Figure 3B). These unique forms of biosynthetic regulation in glycosaminoglycans could be possible targets for the development of novel cancer therapies, but further research is needed in order to achieve it.

## 9. Conclusions and Further Perspectives

Accumulating evidence of glycosaminogylcans now indicates that they actively regulate tumor biology by functioning as signaling co-receptors, modulators of immune responses, and determinants of the physical properties of the tumor microenvironment. As summarized here, heparan sulfate, chondroitin sulfate, and keratan sulfate each possess unique structural characteristics that enable distinct interactions with growth factors, cytokines, chemokines, and cell adhesion molecules, and these factors thereby influence multiple hallmarks of cancer.

Despite these advances, several fundamental questions remain unanswered. One of the most intriguing questions is how specific core proteins are selected for modification with individual glycosaminoglycans. In addition, the molecular mechanisms determining whether the common linker tetrasaccharide is elongated into heparan sulfate or chondroitin sulfate remain poorly understood. Similar questions exist for the biosynthetic predominancy between keratan sulfate and *N*-glycans. Furthermore, although sulfation patterns have long been recognized as critical determinants of ligand binding, the extent to which sulfation functions as a “biological code” governing cellular behavior mostly remains elusive. Addressing these questions will require integrated approaches combining glycobiology, structural biology, chemical biology, systems biology, and advanced glycomics technologies.

From a therapeutic perspective, glycosaminoglycans represent attractive molecular targets because they are positioned at the interface between cancerous cells and their microenvironment. Since glycosaminoglycans express on cell surfaces, they have the ability to simultaneously regulate multiple signaling pathways, cell adhesion, immune responses, and extracellular matrix organization. Therefore, therapeutic modulation of glycosaminoglycans could intracellularly complement targeted therapies. Unfortunately, glycosaminoglycan-targeted therapies are still in the early stages of development. Reported inhibitors for glycosaminoglycans are not specific to cancer cells and would be toxic to normal cells. Additionally, the therapeutic advantage of glycosaminoglycan-targeted CAR-T therapy has not been demonstrated in humans. However, researchers expect future cancer therapies to include selective inhibition of glycosaminoglycan biosynthesis, remodeling of sulfation patterns, enzymatic editing of the glycocalyx, glycomimetic compounds, and glycosaminoglycan-targeted drug delivery systems. Furthermore, by combining glycosaminoglycan-targeted approaches with an immune checkpoint blockade, either CAR-T therapy or molecular-targeted therapies could provide synergistic anti-tumor effects and improve treatment outcomes.

There remain several methodological limitations to analyzing glycosaminoglycans, including determining their chain length and sulfation pattern and identifying core proteins in tumor tissues. Only a few lectins and antibodies are available for analysis, and high-performance liquid chromatography (HPLC) and mass spectrometry analysis remain technically challenging. However, as analytical methods such as high-resolution glycomic analysis, spatial glycomics, single-cell glycomics, and AI-assisted structural prediction continue to improve, our understanding of glycosaminoglycan biology is expected to expand dramatically. These technological advances will facilitate the identification of disease-specific glycan structures in the near future and enable the development of precision glycan-targeted therapeutics in a few decades. We anticipate a time in the near future when deciphering the complex regulatory networks of glycosaminoglycans will not only deepen our understanding of cancer biology but will also provide innovative strategies for diagnosis, prognosis, and treatment.

The functional area, associated or related molecules, and actions of each glycosaminoglycan are listed in Table 1.

## 10. Methods for Searching Studies

The studies introduced in this article were searched using PubMed. To focus on relatively new studies, we mainly selected studies published later than 2000. The search included the keywords glycosaminoglycans, heparan sulfate, chondroitin sulfate, keratan sulfate, and tumor malignancy and was limited to English- and peer-reviewed publications. Primary mechanistic studies, clinical studies, and review articles are included, and the content selectively focuses on the knowledge and concerns of authors working in related fields of study.

## Figures and Tables

**Table 1:** Summary of functional areas, associated or related molecules, and actions of glycosaminoglycans described in this article.

Glycosaminoglycan	Main Functional Areas	Associated or Related Molecules	Actions	References
HS	General biological systems	FGF2, HGF, IL-8, Wnt	Co-receptor activity is involved in malignant tumor phenotypes.	[18,19,20,21]
HS	Glioblastoma	PDGFB, PDGFRα	HS captures PDGFB at the cell surface and potentiates PDGFRα signaling.	[29]
HS	Endothelial cells	VEGF, VEGFR	HS binds VEGF and potentiates the angiogenesis by regulating VEGFR signaling.	[25,26,27]
HS	Endothelial cells	FGF2, FGFR, extracellular histones	HS enhances FGFR signaling to avoid FGF2 degradation, protects the cells from the toxicity of extracellular histons, and contributes to angiogenesis.	[101,102]
HS	Cancer-derived exosomes, NK cells and MDSCs	CCL2, CCR2	HS on exosomes captures CCL2 and potentiates cell signaling via CCR2 in immune cells in the tumor microenvironment.	[28]
HS	Cancer-associated fibroblasts (CAFs)	MMP-7, TGF-β receptors, SULF1/2	HS in CAFs inhibits tumor growth via suppression of MMP-7. SULF1/2 remodels HS and influences tumor signaling.	[98,99,100]
HS	Macrophages	LDLs, type I interferon	HS helps with the uptake of LDLs and is required to maintain type I interferon signaling.	[105,106]
HS	Tumor stroma	CAR-T	HS functions as a physical shield against CAR-T.	[114]
CS	General biological systems	FGF2, VEGF, FGFR, VEGFR	Potential co-receptor activity is involved in malignant tumor phenotypes.	[49,50]
CS	Macrophages	LPS, hyaluronan fragments	CS inhibits NF-κB responses.	[107]
CS-C	Keratinocytes	EGF, EGFR	CS inhibits EGFR signaling and suppresses inflammation; it is potentially involved in regulation of the tumor microenvironment.	[41]
CS-E	General biological systems	FGF2, BDNF, IL-10, Midkine	High-affinity binding to the molecules contributes to co-receptor activity and contributes to cancer signaling.	[37,38,39,40]
Oncofetal chondroitin sulfate (ofCS)	Colorectal, lung, pancreatic, hepatic and prostate cancers	VAR2CSA, integrin, STAT3/4, IL-17/23, CCL5	ofCS is required for integrin signaling and is involved in the immune signal regulation in tumor tissues.	[60,61,62,63,64,65,66]
KS	Pancreatic cancer, melanoma, astrocytic tumor, ovarian cancer	FGF2, IGFBP2, SHH	Potential co-receptor activity; cancer-specific KS would be related to cancer development.	[75,76,77,78,79]
KS	Melanoma	Integrins	KS promotes integrin functions and enhances invasion activity.	[76]
KS core proteins; lumican, podocalyxin	Melanoma, breast, and pancreatic cancer	MMP-14, p120 catenin, ezrin, galectin-3	Core proteins regulate migration, invasion and metastasis; KS-chain-specific contribution is incompletely resolved.	[80,81,82,83,84,85,86]
KS disaccharide L4	Dendritic cells	Langerin, CapG, TNF-α	Anti-inflammatory function of L4 via langerin-CapG-TNF-α axis could be involved in tumor immunity.	[91,92]
GAG-rich glycocalyx	Mesenchymal-like glioblastoma	Integrin, FAK	GAG-rich tumor tissue is mechanically harder and activates integrin-FAK signaling that enhances migration and invasion activity.	[112]
HS versus CS	Glioma	CSGALNACT1, Syndecan-1	Biosynthetic switching from HS to CS on syndecan-1 confers resistance to ferroptosis.	[121]
KS versus *N*-glycan	General cellular glycan biosynthesis	B3GNT7, ST3GALs, ST6GALs	Competition determines whether *N*-glycan is elongated into KS or capped with sialic acid.	N/A

## Data Availability

No new data are included in the manuscript.

## Abbreviations

AMPK, AMP-activated protein kinase; B4GNT7, β1,3-*N*-acetylglucosaminyltransferase 7; BDNF, brain-derived neurotrophic factor; CA19-9, carbohydrate antigen 19-9; CAF, cancer-associated fibroblast; CapG, macrophage-capping protein; CAR, chimeric antigen receptor; CAR-T, chimeric antigen receptor T-cell; CCL2, C-C motif chemokine ligand 2; CCL5, C-C motif chemokine ligand 5; CCR2, C-C motif chemokine receptor 2; CEA, carcinoembryonic antigen; CS, chondroitin sulfate; CSGALNACT, chondroitin sulfate *N*-acetylgalactosaminyltransferases; CSPG, chondroitin sulfate proteoglycan; EGFR, epidermal growth factor receptor; EMT, epithelial–mesenchymal transition; EXT, exostosin; EXTL, exostosis-like; FAK, focal adhesion kinase; FGF2, fibroblast growth factor 2; FGFR, fibroblast growth factor receptor; GAGs, glycosaminoglycans; GD2, ganglioside GD2; GlcNAc, *N*-acetylglucosamine; GPI, glycosyl–phosphatidyl–inositol; HGF, hepatocyte growth factor; HPLC, high-performance liquid chromatography; HS, heparan sulfate; HS6ST, heparan sulfate 6-*O*-sulfotransferase; IGFBP2, insulin-like growth factor binding protein 2; IL-8, interleukin-8; IL-10, interleukin-10; IL-17, interleukin-17; IL-23, interleukin-23; JAK/STAT, Janus kinase/signal transducer and activator of transcription; KS, keratan sulfate; LPS, lipopolysaccharide; MAPK, mitogen-activated protein kinase; MDSCs, myeloid-derived suppressor cells; MMP, matrix metalloproteinase; NF-κB, nuclear factor kappa B; NK, natural killer; NS, sulfation at the *N*-acetyl group; PDGFB, platelet-derived growth factor B; PDGFRα, platelet-derived growth factor receptor α; PI3K, phosphatidylinositol 3-kinase; PTPR, protein tyrosine phosphatase receptor type σ; SHH, sonic hedgehog; Sia, sialic acid; sLe^a^, sialyl Lewis a; STAT, signal transducer and activator of transcription; SULF, heparan-6-*O*-endosulfatase; TGF-β, transforming growth factor-β; TGFBR3, transforming growth factor-β receptor III; TNF-α, tumor necrosis factor-α; VEGF, vascular endothelial growth factor; VEGFR, vascular endothelial growth factor receptor; Wnt, wingless-related integration site; Xyl, xylose.
